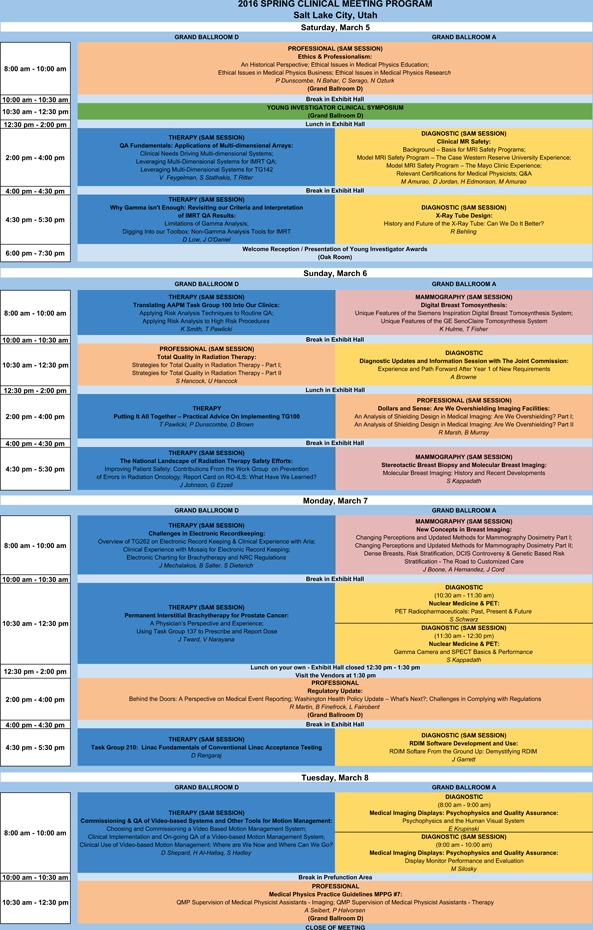# 2016 AAPM Spring Clinical Meeting March 5 ‐ 8, 2016 Salt Lake City, UT

**DOI:** 10.1120/jacmp.v17i3.6471

**Published:** 2016-05-04

MEETING PROGRAM

*Available on‐line at* www.aapm.org/meetlngs/2016SCM/

Chair

Jessica B. Clements, MS

Kaiser Permanente Los Angeles, CA

Vice Chair

Michael Howard, PhD

Sarah Cannon Cancer Center Chattanooga, TN

**Track Directors**

**Therapy Track**

Jean M. Moran, PhD

University Michigan Medical Center Ann Arbor, MI

Kyle J. Antes, MS

Presbyterian Healthcare System Dallas, TX

Brian Wang, PhD

University Louisville Louisville, KY

**Professional Track**

Michael Howard, PhD

Sarah Cannon Cancer Center Chattanooga, TN

Brent C. Parker, PhD

**University Texas Medical Branch of Galveston Galveston, TX**

**Diagnostic Track**

Dustin Gress, MS

**MD Anderson Cancer Center Houston, TX**

Jeffrey M. Moirano, MS

**University of Washington Seattle, WA**

**Mammography Track**

Jessica B. Clements, MS

**Kaiser Permanente Los Angeles, CA**

**Young Investigator Program**

Jean M. Moran, PhD

**University Michigan Medical Center Ann Arbor, MI**

Jeffrey M. Moirano, MS

**University of Washington Seattle, WA**

**Figure 1:**